# The management of displaced intracapsular femoral neck fractures at a Sub-Saharan Academic Hospital

**DOI:** 10.1051/sicotj/2021023

**Published:** 2021-05-19

**Authors:** Winifred Mukiibi, Zia Maharaj, Allan Roy Sekeitto, Lipalo Mokete, Jurek Rafal Tomasz Pietrzak

**Affiliations:** 1 Arthroplasty Unit, Department of Orthopaedics, Charlotte Maxeke Johannesburg Hospital, University of Witwatersrand 2000 Johannesburg South Africa

**Keywords:** Intracapsular femoral neck fractures, Sernbo score, Total hip arthroplasty, Hemiarthroplasty

## Abstract

*Background*: Femoral neck fractures (FNFs) remain “the unsolved fracture” and optimal management is still controversial. The outcomes of hemiarthroplasty (HA) and total hip arthroplasty (THA) in the treatment of FNFs are inconsistent. As demand for management of FNFs continues to grow globally, evaluation of the appropriateness of treatment remains essential, particularly in resource-constrained settings. *Methods*: We conducted a retrospective chart review of all patients presenting with isolated low energy intracapsular FNFs to an orthopaedic academic unit in Sub-Saharan Africa from January 2016 to April 2019. The decision regarding HA or THA was largely based upon the Sernbo score and ASA classification. The majority of patients with a Sernbo score of ≥15 and ASA class III or better received THA. *Results*: There were 117 patients (33 male/84 female) 72 years (33–97 years) with FNFs who underwent 56 THA and 61 HA between January 2016 and April 2019. The mean Sernbo score was 15.99 overall (range 8–20) and was 18.95 (11–20) for THA patients compared to 14.46 (8–20) for HA patients (*p* = 0.042). Time taken from admission to the theatre was 8–19 days (1–22) and 7–61 days (2–31) for HA and THA respectively. The average length of stay (LOS) was 16.04 days and the main reason for same-day cancellations was the lack of post ICU/High Care beds. The 30-day mortality rates were 1.78% and 4.91% for THA and HA patients, respectively (*p* = 0.07). The mortality rate for patients with a Sernbo score < 15 was 15.38% overall, 8.93% for THA patients, and 21.31% for HA patients, respectively (*p* = 0.021). *Conclusion*: The 30-day mortality rate was comparable with published rates from developed countries. There were significant delays in time to theatre, high rates of same-day surgical cancellations, and increased LOS for both HA and THA. These factors play a cumulative role in inflating costs on a strained healthcare system in a developing country. A multidisciplinary approach including the care provision of a specialized geriatric unit is recommended.

Retrospective Study, Level III evidence

## Introduction

Each year, approximately 1.3 million femoral neck fractures (FNFs) occur worldwide [1, 2]. The incidence of FNFs globally is increasing [1, 3] and is expected to exceed 7.3 million patients around the world by 2050 [4]. In the United Kingdom (UK) it is estimated that there will be almost 100,000 FNFs in 2020 alone [1, 5]. The financial repercussion of managing FNFs in the UK will be as much as 2 billion pounds annually [1, 5] and costs will likely increase with time. There is a paucity of published articles on FNF emanating from the developing world. However, there are greater constraints in access to healthcare and resources in developing countries [6] likely making FNF treatment a challenge.

Optimal management of FNFs remains controversial [7]. Surgical treatment options for displaced intracapsular FNFs include total hip arthroplasty (THA) and hemiarthroplasty (HA) and are dependent on several factors including patient age and activity level [7, 8]. Conservative management is appropriate in a minority of patients as a consequence of pre-existing medical conditions, negligible pre-morbid ambulatory capability, and objectives of future care [9]. Superior functional outcome scores [7] and more significant pain amelioration [8] have been reported for FNFs treated surgically with THA as opposed to HA.

The irrespective ideal treatment of FNFs should preserve life, be cost-effective, and limit the need for post-morbid social housing [1]. However, even with the best care, mortality rates as a result of FNFs remain alarming: 6% of patients demise before reaching hospital; 10% within 30 days [1] and up to 36% of patients demise within 1 year of injury [10]. The first 6 months post-FNF represents the time period of greatest risk for mortality [11].

The ideal time to treatment of FNFs remains contentious [12–20]. Throughout the world, most national guidelines recommend “early” surgical intervention preferably within 48 h of admission [12–20]. A balance needs, however, to be achieved between early surgery and avoiding immobility, thrombosis, and pressure sores while still allowing sufficient time to optimize the patients’ clinical condition and potentially improve all reversible co-morbidities [1].

Our institution is based in an urban sub-Saharan African city. The primary aim of this study was, therefore, to determine the outcomes of the management of displaced intracapsular FNFs, including complication, readmission, and mortality rates, of patients treated at an academic referral centre in a developing country. Secondarily, we sought to evaluate the impact that patient-specific factors such as age, gender, Sernbo score, and institutional-related issues including time to surgery, time to discharge, and surgical cancellations influenced the complication and mortality rate.

## Materials and methods

### Study design

This was a retrospective chart review of 125 consecutive adult patients admitted to an urban South African hospital. Ethics approval was granted by the institutional review board Human Research Ethics Council (HREC) of the University of Witwatersrand (M190477).

### Patients

The study included all consecutive patients older than 18 years, that presented with isolated, displaced, low-energy intracapsular femoral neck fractures (FNFs) from January 2016 to April 2019 with a minimum follow-up of 2 years. We excluded patients presenting with undisplaced FNFs extracapsular FNFs, patients who refused consent to participate in the study, and patients with multiple injuries including concurrent ipsilateral femur fractures.

On admission, all patients were evaluated and stabilised in the emergency triage department before being referred to the Orthopaedic Department. The diagnosis of a displaced, intracapsular FNF was made on routine anterior–posterior (AP) pelvis and lateral hip X-rays. Displaced FNFs were further classified according to Garden Classification. There is no specialised geriatric service at our institution and at the time of the study, there was no formal protocol for the surgical management of patients with displaced FNFs. All patients in the current study were, however, planned for surgical intervention. The choice between THA and HA was individualized and made on a case-by-case basis by a collaborative effort of consultants from both the Trauma and Arthroplasty services at a combined post-admission department meeting. The Trauma service does all HAs and THAs are done by the Arthroplasty service at our institution.

We had been using the ASA classification [21] as a guide to decision-making in FNF treatment prior to 2016. Only patients with a minimum grade III classification were considered for THA. However, we recognized that the ASA classification on its own was inadequate to allow us to come to a consistent conclusion regarding the choice between HA and THA in a significant number of patients.

In 2016, the Orthopaedic unit made a decision to adopt the Sernbo score as a guide to help refine decision making on the basis of simplicity of the score and evidence showing the score was not only helpful in deciding between HA and THA [22] but also predicted survival in FNF [23]. We found other established scoring systems to be too complicated and impractical to apply in our resource-poor setting [24, 25]. We considered patients with a Sernbo score of ≥15 and ASA class III candidates for THA. Our population included younger patients, therefore, we modified the age aspect of the original Sernbo score awarding a score of 5 for age 80 years or less and 2 for age more than 80 years. We erred on the side of THA in patients that were younger than 60 years with uncompromised pre-morbid activity levels.

### Surgical procedure

All HAs were performed by a single consultant surgeon who is arthroplasty trained. All THAs were performed by or under the supervision of one of three experienced Arthroplasty-trained consultants. All patients were given prophylactic intravenous antibiotics and tranexamic acid (TXA) 30–60 min prior to the first surgical incision. Prophylactic antibiotics included a weight-adjusted dose of first-generation cephalosporin or clindamycin in penicillin-allergic patients. The surgical approach was a modified anterolateral approach for all HA and a modified anterolateral or posterior approach for all THA. Surgical implants and fixation techniques (cemented or uncemented) were recorded for all patients.

Post-operatively, haemoglobin (Hb) levels were assessed in all patients. The transfusion trigger was a Hb of less than 8 g/dL and symptomatic patients with a higher Hb level were transfused based on the discretion of the treating surgeon. Post-op X-rays were taken and assessed by surgeons. Mobilisation was initiated by physiotherapists on day 1 post-operatively. Patients were followed up at 2 weeks, one month, 6 months, one year, and two years postoperatively. Missed appointments were followed up with telephonic contact.

### Main variables and outcome measures

All data was captured into a standardized data collection form. This included demographic details, mechanism of injury (MOI), medical co-morbidities, American Society of Anesthesiologists (ASA) classification, the time between hospital admission and surgery(days); length of hospital stay (days); procedure performed; and inpatient complications. The Sernbo score was calculated by the admitting doctor and confirmed by the arthroplasty service.

Hospital notes were analysed to determine peri-operative complications. Complications were classified as intra-operative and post-operative. Post-operative complications were further divided into medical and surgical complications. The impact of both patient-specific factors and institution-related issues on the overall mortality rate was evaluated. Patient-specific factors included the patient’s age (<65 and >66 years of age); gender (male or female); ASA class; Sernbo score (<15 and ≥15). The institution-related factors included the number of days from admission to the theatre (>7, 8–14 and >15 days respectively); the time from theatre to discharge (>7, 8–14 and >15 days respectively), and the number of surgical cancellations (0, 1 and ≥2 cancellations respectively).

### Statistical analysis

The data was tabulated and the results were compared between patients for THA and HA. Students’ *t*-tests and chi-squared testing was used to compare integer and categorical variables, respectively. Predictors of mortality were compared between patients for THA and HA using chi-squared testing. Statistical significance was set at *p* < 0.05. Software used for analysis was R 4.0.2 for Windows Copyright (C) 1989, 1991 Free Software Foundation, Inc with interface R Studio Version 1.3.959.

## Results

**Figure 1:**
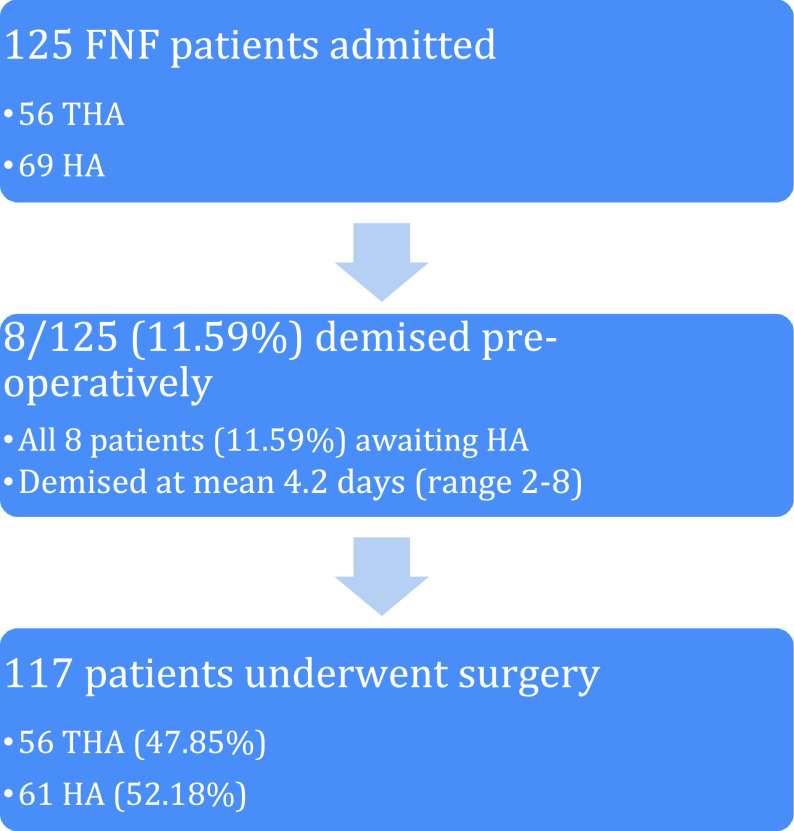
Study cohort. FNF = femoral neck fracture; THA = total hip arthroplasty; HA = hemiarthroplasty.

We included 117 patients (93.60%) in the study from 125 patients that were admitted and eligible for participation (Figure 1). The mean age was 72 years ± 13.24 and 71.78% (*n* = 84) of patients were female (Table 1). The mean age of patients for THA was 66.95 years and it was 76.64 years for HA (*p* = 0.046). The mean Sernbo score was 15.99 overall (range 8–20) and was 18.95 for THA patients compared to 14.46 for HA patients (*p* = 0.042). The overall incidence of dementia was 17.94% (*n* = 21) and it was 8.92% (*n* = 5) for THA patients compared to 26.22% (*n* = 16) for HA patients (*n* = 0.036).

**Table 1:** Demographic details for total population (*n* = 117) and demographic data of patients for total hip arthroplasty (*n* = 56) compared to those for hemiarthroplasty (*n* = 61).

Demographic	Total	THA	HA	*P*-value
Age (years), mean ± SD (range)	72 ± 13.24 (33–97)	66.95 ± 11.89 (33–88)	76.64 ± 12.80 (47–97)	0.046
Gender, *n* (%)				
Male	33 (28.22)	16 (28.57)	17 (27.87)	0.898
Female	84 (71.78)	40 (71.43)	44 (72.13)	
Race, *n* (%)				
Black	31 (26.49)	16 (28.57)	15 (24.59)	0.043
Caucasian	63 (53.84)	34 (60.71)	29 (47.54)	
Indian descent	9 (7.69)	4 (7.14)	5 (8.19)	
Mixed race	12 (10.25)	2 (3.57)	10 (16.39)	
Other	2 (1.70)	0 (0)	2 (3.27)	
Mechanism of injury, *n* (%)				
FFSH	90 (76.92)	41 (73.21)	49 (80.32)	0.66
MVA	16 (13.67)	11 (19.64)	5 (8.19)	
PVA	11 (9.40)	4 (7.14)	7 (11.47)	
Sernbo score, mean (range)	15.99 (8–20)	18.95 (11–20)	14.46 (8–20)	0.042
Functionality, *n* (%)				0.048
Community walker	85 (72.64)	53 (94.64)	32 (52.45)	
Domestic walker only	32 (27.35)	3 (5.35)	29 (47.54)	
Mental state, *n* (%)				0.036
Alert to PP and T	96 (82.05)	51 (91.07)	45 (73.77)	
Dementia	21 (17.94)	5 (8.92)	16 (26.22)	
Living situation, *n* (%)				0.282
Own home	87 (74.35)	49 (87.50)	38 (62.29)	
Old age home	19 (16.23)	5 (8.92)	14 (22.95)	
Institution	4 (3.41)	0 (0)	4 (6.55)	
Care facility	7 (5.98)	2 (3.57)	5 (8.19)	
Walking aid, *n* (%)				0.215
None	72 (61.53)	49 (87.50)	23 (37.70)	
Cane	11 (9.40)	0 (0)	11 (18.03)	
1 crutch	3 (2.56)	0 (0)	3 (4.91)	
2 crutches	12 (10.25)	3 (5.35)	9 (14.75)	
Walking frame	9 (7.69)	2 (3.57)	7 (11.47)	
Rollator	10 (8.54)	2 (3.57)	8 (13.11)	
ASA class, *n* (%)				0.471
1	74 (63.24)	43 (76.78)	31 (50.08)	
2	34 (29.05)	11 (19.64)	23 (37.70)	
3	9 (7.69)	2 (3.57)	7 (11.47)	
Number comorbidities, *n* (%)				0.86
0	34 (29.05)	17 (30.35)	17 (27.86)	
1	39 (33.33)	26 (46.42)	13 (21.31)	
2	25 (21.36)	11 (19.64)	14 (22.95)	
3	11 (9.40)	0 (0)	11 (18.03)	
≥4	8 (6.83)	2 (3.57)	6 (9.83)	
Comorbidities, *n* (%)				0.747
Diabetes	13 (13.83)	7 (14.58)	6 (13.04)	
Hypertension	44 (46.81)	22 (45.83)	22 (47.83)	
Epilepsy	7 (7.45)	4 (8.33)	3 (6.52)	
Rheumatoid arthritis	2 (2.13)	0 (0)	2 (4.34)	
Ischaemic heart disease	8 (8.51)	3 (6.25)	5 (10.86)	
Cardiac arrhythmias	6 (6.38)	4 (8.33)	2 (4.34)	
CCF	6 (6.38)	0 (0)	6 (13.04)	
Asthma	1 (1.06)	1 (2.08)	0 (0)	
COPD	6 (6.38)	3 (6.25)	3 (6.52)	
RVD	5 (5.32)	3 (6.25)	2(4.34)	
CVA	11 (11.70)	4 (8.33)	7 (15.21)	

THA = total hip arthroplasty; HA = hemiarthroplasty; SD = standard deviation; FFSH = Fall from standing height; MVA = motor vehicle accident; PVA = pedestrian vehicle accident; PP and T = person, place and time; ASA Class = American College of Anaesthesiologists Classification; CCF = congestive cardiac failure; COPD = chronic obstructive pulmonary disease; RVD = Retroviral Disease; CVA = cerbrovascular accident.

The overall mortality rate was 26.50% (*n* = 31), 6.84% (*n* = 8) for pre-operative mortality and 19.66% (*n* = 23) for the post-operative mortality (Table 2). The pre-operative mortality rate was 13.11% (*n* = 8) for planned HA patients while no planned THA patients demised prior to surgery (*p* = 0.023). The 30-day mortality rates were 1.78% and 4.91% for THA and HA patients, respectively (*p* = 0.07). Similarly, the 90-Day mortality rates were 3.57% and 6.55% for THA and HA patients, respectively (*p* = 0.08).

**Table 2:** Readmissions, complications and mortality rates for total population (*n* = 117) and of patients for total hip arthroplasty (*n* = 56) compared to those for hemiarthroplasty (*n* = 61).

Mortality rate	Total, *n* (%)	THA, *n* (%)	HA, *n* (%)	*P*-value
Overall mortality	31 (26.50)	6 (10.71)	25 (40.98)	0.036
Pre-operative	8 (6.84)	0 (0)	8 (13.11)	0.023
Post-operative	23 (19.66)	6 (10.71)	17 (27.87)	0.041
<30 days	10 (8.55)	1 (1.79)	9 (14.75)	
31–60 days	3 (2.56)	2 (3.57)	1 (1.64)	
61–90 days	4 (3.42)	2 (3.57)	2 (3.28)	
3–6 months	6 (5.13)	1 (1.79)	5 (8.20)	
Readmission rate				
30-day	4 (4.25)	1 (1.78)	3 (4.91)	0.07
60-day	7 (7.45)	3 (5.35)	4 (6.55)	0.78
90-day	7 (7.45)	3 (5.35)	4 (6.55)	0.08
PJI	2 (2.56)	1 (1.78)	1 (1.63)	
Dislocation	0 (0)	1 (1.78)	0 (0)	
DVT	3 (2.56)	1 (1.78)	2 (3.27)	
Implant failure	1 (0.85)	0 (0)	1 (1.63)	
Complication rate				
Total	21 (17.95)	7 (12.50)	14 (22.95)	0.21
Medical				0.891
Overall	14 (11.97)	3 (5.36)	11 (18.03)	
DVT	3 (2.56)	1 (1.79)	2 (3.28)	
MI	5 (4.27)	1 (1.79)	4 (6.56)	
PE	1 (0.85)	0 (0)	1 (1.64)	
AKI	5 (4.27)	2 (3.57)	3 (4.92)	
Surgical				0.69
Overall	7 (5.98)	4 (7.14)	3 (4.92)	
Intra-operative	3 (2.56)	2 (3.57)	1 (1.64)	
Post-operative	4 (3.42)	2 (3.57)	2 (3.28)	
PJI	2 (1.71)	1 (1.79)	1 (1.64)	
Dislocation	1 (0.85)	1 (1.79)	0 (0)	
Implant failure	1 (0.85)	0 (0)	1 (1.64)	

THA = total hip arthroplasty; HA = hemiarthroplasty; DVT = deep vein thrombosis; PE = pulmonary embolism; AKI = acute kidney injury; PJI = periprostethic joint infection; SSI = superficial skin infection; UTI = urinary tract infection.

**Table 3:** Risk factors associated with mortality for total population (*n* = 117) and compared between patients for total hip arthroplasty (*n* = 56) and hemiarthroplasty (*n* = 61).

Patient factor	Mortality rate, *n* (%)			*P*-value
	Total	THA	HA	
Age (years)				0.031
<65	7 (5.98)	4 (7.14)	3 (4.92)	
>65	16 (13.68)	2 (3.57)	14 (22.95)	
Gender				0.36
Male	4 (3.42)	2 (3.57)	2 (3.28)	
Female	19 (16.24)	4 (7.14)	15 (24.59)	
ASA class				0.45
1	8 (6.84)	1 (1.79)	7 (11.48)	
2	7 (5.98)	3 (5.36)	4 (6.56)	
3	8 (6.84)	2 (3.57)	6 (9.84)	
Sernbo score				0.021
<15	18 (15.38)	5 (8.93)	13 (21.31)	
≥15	5 (4.27)	1 (1.79)	4 (6.56)	
Mental state				0.69
Alert to PP and T	12 (10.26)	3 (5.36)	9 (14.75)	
Dementia	11 (9.40)	3 (5.36)	8 (13.11)	
Admission to theatre (days)				0.032
<7	1 (0.85)	0 (0)	1 (1.64)	
8–14	5 (4.27)	4 (7.14)	1 (1.64)	
>15	17 (14.53)	2 (3.57)	15 (24.59)	
Theatre to discharge (days)				0.436
<7	7 (5.98)	1 (1.79)	6 (9.84)	
8–14	10 (8.55)	3 (5.36)	7 (11.48)	
>15	6 (5.13)	2 (3.57)	4 (6.56)	
Number of cancellations				0.042
0	2 (1.71)	1 (1.79)	1 (1.64)	
1	5 (4.27)	3 (5.36)	2 (3.28)	
>2	16 (13.68)	2 (3.57)	14 (22.95)	

THA = total hip arthroplasty; HA = hemiarthroplasty; PP and T = person, place and time; ASA Class = American College of Anaesthesiologists Classification.

**Table 4:** Hospital inpatient details for total population (*n* = 117) and data of patients for total hip arthroplasty (*n* = 56) compared to those for hemiarthroplasty (*n* = 61).

	Total	THA	HA	*P*-value
Peri-operative time frame (days), mean ± SD				
Admission to theatre	7.83 ± 5.77	7.48 ± 6.46	8.19 ± 4.99	0.032
Theatre to discharge	8.12 ± 10.47	7.36 ± 10.94	8.67 ± 10.28	0.436
Length of hospital stay	16.27 ± 12.98	14.05 ± 13.56	17.90 ± 12.51	0.032
Cancellations, *n* (%)				0.812
Total	38 (32.48)	18 (32.14)	20 (32.79)	
No ICU bed available	18 (15.38)	11 (19.64)	7 (11.48)	
No HCA bed available	3 (2.56)	1 (1.79)	2 (3.28)	
Need echocardiogram	7 (5.98)	3 (5.36)	4 (6.56)	
New hypertension	1 (0.85)	0 (0)	1 (1.64)	
INR > 1.5	3 (2.56)	1 (1.79)	2 (3.28)	
PE	1 (0.85)	0 (0)	1 (1.64)	
On anticoagulant therapy	1 (0.85)	0 (0)	1 (1.64)	
Theatre time constraints	3 (2.56)	2 (3.57)	1 (1.64)	

THA = total hip arthroplasty; HA = hemiarthroplasty; SD = standard deviation; ICU = intensive care unit; HCA = high care area; INR = international normalised ratio; PE = pulmonary embolism.

The risk factors associated with mortality for the study population were compared between patients for THA and HA (Table 3). The mortality rate for patients older than 65 years was 13.68% (*n* = 16) overall and 3.57% (*n* = 2) and 22.95% (*n* = 14) for THA and HA patients, respectively (*p* = 0.031). The mortality rate for patients with a Sernbo score < 15 was 15.38%, 8.93% for THA patients, and 21.31% for HA patients (*p* = 0.021). The mortality rate for patients awaiting surgery for longer than 15 days was 3.57% (*n* = 2) for THA patients compared to 24.59% (*n* = 15) for HA patients (*p* = 0.032). Similarly, the mortality rate for patients who had their surgery cancelled more than 2 times was 3.57% (*n* = 2) for THA patients and 22.95% (*n* = 14) for HA patients (*p* = 0.042).

There was a higher incidence of medical compared to surgical complications. There were 14 (11.97%) medical and 7 (5.98%) surgical complications. The incidence of medical complications was 5.36% and 18.03% for THA and HA patients, respectively (*p* = 0.891). The surgical complication rate was 7.14% and 4.92% for THA and HA patients, respectively (*p* = 0.69).

The details for the course of hospital stay of all admitted patients are shown in (Table 4). The overall mean time was 7.83 days (range 1–31) from admission to surgical intervention and 8.12 days (range 5–54) from surgery to discharge, respectively. The mean length of hospital stay was 16.24 days ± 12.98 (range 5–71) for the study population. The mean time from admission to theatre for THA and HA patients was 7.48 and 8.19 days, respectively (*p* = 0.032). Similarly, the mean length of hospital stay for THA and HA patients was 14.05 and 17.90 days, respectively (*p* = 0.032). There was a total of 38 (32.48%) surgery cancellations.

## Discussion

FNFs remains an epidemic of orthopaedic concern with increasing numbers presenting globally in both developing and developed countries [26]. Downey et al. searched systematic literature reviews, national hip fracture registries, databases, and local studies pertaining to hip fractures from the English-speaking literature [26]. They discovered work from 36 countries internationally, but none from Africa in the last 5 years [26]. Additionally, Kanis et al. revealed that a 10-fold variation in the incidence of FNF based solely on a single geographic location alone [27]. An African perspective, therefore, remains essential.

### Mortality

In this study from a Sub-Saharan African academic institution, the overall 30-day mortality rate of displaced intra-capsular FNF was 8.01%. This compares favourably with the 30-day mortality rate from developed countries reported by Johansen and Lewis [28, 29]. Similarly, our in-hospital mortality rate of 8.51% compares favourably to the 7% reported by Sheehan et al. [30]. A significant difference in the 30-day mortality rate existed between patients treated with HA (13.04%) and THA (1.78%) (*p* = 0.08). This is also comparable with other reported studies. In a review of 43,891 patients, Nemes et al. reported that HA inferred a worse survival rate than THA (55% vs. 87.5%) [31]. Liodakis et al. found a higher mortality rate for HA compared to THA in 4058 patients treated for FNF [32]. In our study, the in-patient, 30-day, 60-day, and 90-day mortality rates were all significantly worse if surgery was performed more than a week after admission (*p* = 0.042).

### Postoperative complications

The overall complication rate in this study was 17.94%. Peri-operative complications after the surgical management of displaced FNFs are common [32–35]. Post-operative, early complications were commoner in HA (22.95%) than in THA (12.5%). There were more medical 14(11.97%) than surgical 7(5.98%) complications in our study in contrast to findings by Mu Heo et al. [36]. This may be attributed to the high rate of pre-existing medical co-morbidities (33.33% with more than 1) with a predominance of cardiovascular disease among patients in our study population. Cardiovascular events including MI are a major cause of morbidity, mortality, and increased LOS in patients undergoing orthopaedic procedures [33].

### Readmissions

The 30-day readmission rate in our study was 4 (4.25%) overall and THA group 1 (1.78%) and HA group 3 (4.91%) Similar to 3.65% reported by Mednick et al. [37]. Jencks et al. in a large cohort of 11,855,702 patients reported a much higher readmission rate of 19.6% [33]. Our overall 60-day readmission rate was 7 (7.45%). We observed that an increased pre-operative co-morbidity burden increased the rate of readmissions [37].

### Length of stay

The average LOS in our study was 16.04 days with no statistically significant difference between THA and HA. This was higher compared to findings by Kat et al., who looked at LOS of a patient with hip fractures and found it to be an average of between 4.08 and 5.55 days [38]. Patient characteristics and hospital resources are factors that contribute to increased LOS [38]. This includes high ASA scores, male gender, and decreased hospital surgical resources over the weekend [38]. Post-operative complications also contribute to increased length of hospital stay (LOS) [32, 34]. However, we believe that factors including lack of institutional support from a specialist geriatric unit, high theatre cancellation rate by anesthetists on the day of planned surgery (32%), limited availability of post-operative intensive/high care beds, and lack of step-down care facilities were the main reasons for increased LOS in our patient cohort.

### Surgical delay

The average time from hospital admission to surgical intervention was 7.9 days and 7.61 days for HA and THA respectively. The degree to which surgical delay adversely impacts the outcomes of FNF management is uncertain but the literature proposes a threshold of 24–48 h [22, 23, 30, 31]. Limiting the waiting time for surgery in FNFs results in improved pain control, decreased complications, accelerated mobilization, and decreased LOS [30, 32–34]. Mortality has been shown to increase by 1, 5% for every hour of surgical delay beyond 24 h [35]. 6.4% of patients demised before surgical intervention at least 48 h following hospital admission. No patients had surgery within 72 h of admission in our study. This was due to surgery for FNFs being done on a semi-elective basis after medical work-up and review by anaesthetists with no weekend and after-hours surgery. Valuable time was lost waiting for medical review and results of investigations. Often surgery was cancelled by anaesthesia on the day of surgery. The main reasons accounting for almost three-quarters of such cancellations being the lack of post-op high-care/ICU beds 21 (55, 26%) and a new request for an echocardiogram 7 (18, 42%). We are uncertain whether surgery in the absence of a high care/ICU bed and echocardiogram would have resulted in worse outcomes, but we recognize that undue delay in surgery is associated with poorer outcomes [30].

In the United Kingdom, the ability to operate on patients with FNFs within 36 h is used as a surrogate marker of the quality of care [39]. In a meta-analysis of 257,367 FNFs, Shiga et al. found that delay of surgery beyond 48 h after admission increased the risk of 30-day mortality and 1-year mortality due to all causes by 41% and 32% respectively [40]. Additionally, this risk was especially acute in low-risk and younger patients [38].

There were several limitations to our study. The data were collected and reviewed retrospectively. The study sample was relatively small reflecting our demographics of a relatively young population and a treatment burden that is skewed towards high-velocity emergency trauma management. Our patient cohort was not limited to elderly patients but included young patients as well. This resulted in possibly a higher proportion of patients receiving THA.

Globally, the management of FNFs may serve as an indicator of the quality of care of the geriatric population [28, 41]. In this study, there were significant delays in time to theatre, high rates of same-day surgical cancellations, and increased LOS. These play a cumulative role in inflating costs on an already strained healthcare system in a developing country. A multidisciplinary approach including the care provision of a specialized geriatric unit is recommended to potentiate the best possible clinical outcomes [30, 42]. This was also emphasized by Mudiganty et al. who described a coordinated team effort, including the emergency doctors, surgeons, and anaesthetic team involvement [41]. Few institutions in developing countries, currently have access to such clinical service provisions [43]. However, the development of local guidelines for the management of displaced FNFs by a multidisciplinary team based on available resources will likely result in significant improvements in quality of care and treatment outcomes.

## Conflict of interest

There is no conflict of interest to declare.
